# Supplementary material for: The p38/MK2 Pathway Functions as Chk1-Backup Downstream of ATM/ATR in G2-Checkpoint Activation in Cells Exposed to Ionizing Radiation
Source: Cells. 2023 May 14;12(10):1387. doi: 10.3390/cells12101387 (PMC10216922; doi:10.3390/cells12101387)
Supplement: Supplementary file 1 [file cells-12-01387-s001.zip › cells-2327335-supplementary.pdf]

Supplementary information

The p38/MK2 pathway functions as Chk1-backup downstream of ATM/ATR in G_2-checkpoint activation in cells exposed to ionizing radiation

Luo et al., Cells

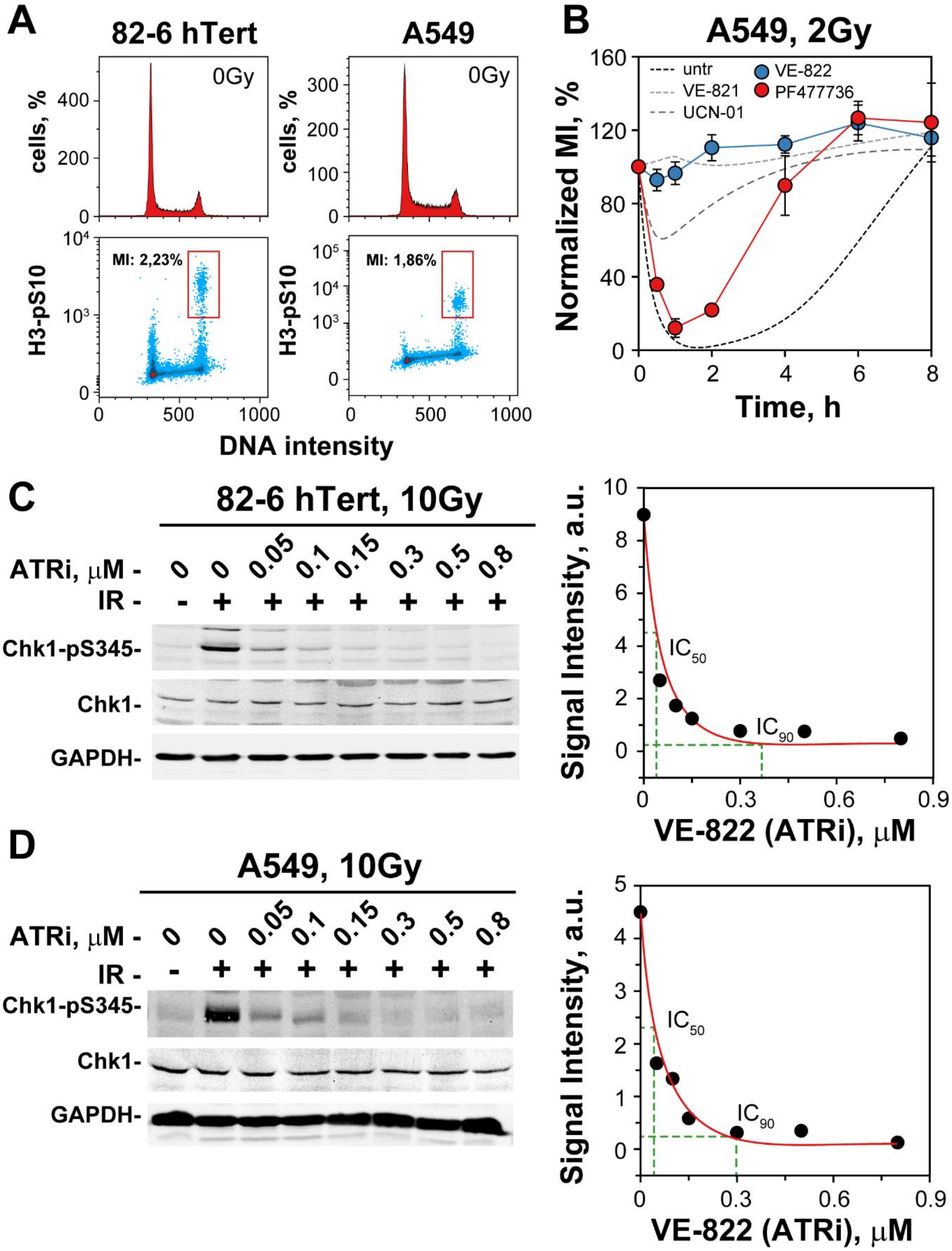
A
82-6 hTert
A549
0Gy
cells, %
MI: 2,23%
MI: 1,86%
H3-pS10
DNA intensity
B
A549, 2Gy
untr
VE-821
UCN-01
VE-822
PF477736
Normalized MI, %
Time, h
C
82-6 hTert, 10Gy
ATRi, μM -
IR -
Chk1-pS345-
Chk1-
GAPDH-
Signal Intensity, a.u.
IC50
IC90
VE-822 (ATRi), μM
D
A549, 10Gy
ATRi, μM -
IR -
Chk1-pS345-
Chk1-
GAPDH-
Signal Intensity, a.u.
IC50
IC90
VE-822 (ATRi), μM

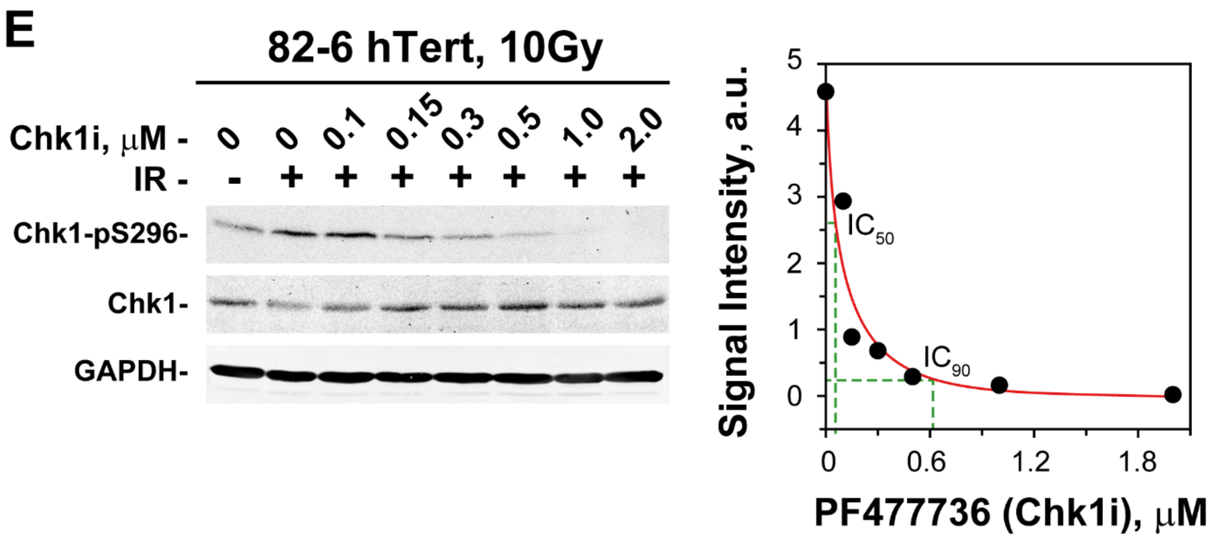

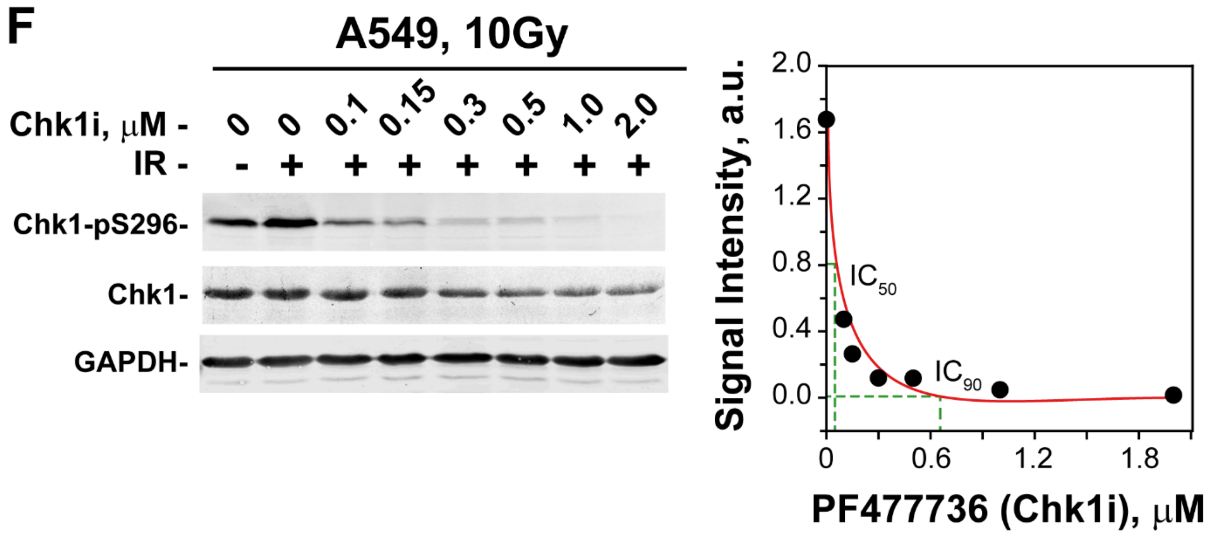

Figure S1. (A) Representative histograms (upper panels) and dot plots (lower panels) illustrating results of two-parameter flow cytometry analysis including DNA content and H3-pS10 signal for the determination of MI in 82-6 hTert and A549 cells. The gates used to select for H3-pS10 positive cells are depicted by red rectangles. **(B)** Normalized MI analysis of A549 cells irradiated with 2 Gy in the presence of ATR inhibitor, VE-822, or Chk1 inhibitor, PF477736. Other details as in Figure 1A. Data represent the mean and standard deviation (± SD) from three independent experiments. **(C)** Western blot analysis showing the effect of increasing concentrations of VE-822, an inhibitor of ATR (ATRi), on 82-6 hTert cells exposed to 10 Gy and analyzed 1 h later. ATR activity is measured using the phosphorylation of its target, Chk1, on Serine 345 (Chk1-pS345) as a proxy. Total Chk1 levels are also shown. GAPDH serves as a loading control. Densitometry analysis of Chk1-pS345 signal is used for calculation of the IC50 and IC90 for ATRi (right panel). **(D)** Same as panel C for A549 cells. **(E)** Western blot analysis showing the effect of increasing

concentrations of PF477736, an inhibitor of Chk1 (Chk1i), on 82-6 hTert cells exposed to 10 Gy and analyzed 1 h later. Chk1 activity is measured using its autophosphorylation on Serine 296 (Chk1-pS296) as a proxy. Total Chk1 levels are also shown. GAPDH serves as a loading control. Densitometry analysis of Chk1-pS296 signal is used for calculation of the IC50 and IC90 for Chk1i (right panel). **(F)** Same as panel E for A549 cells.

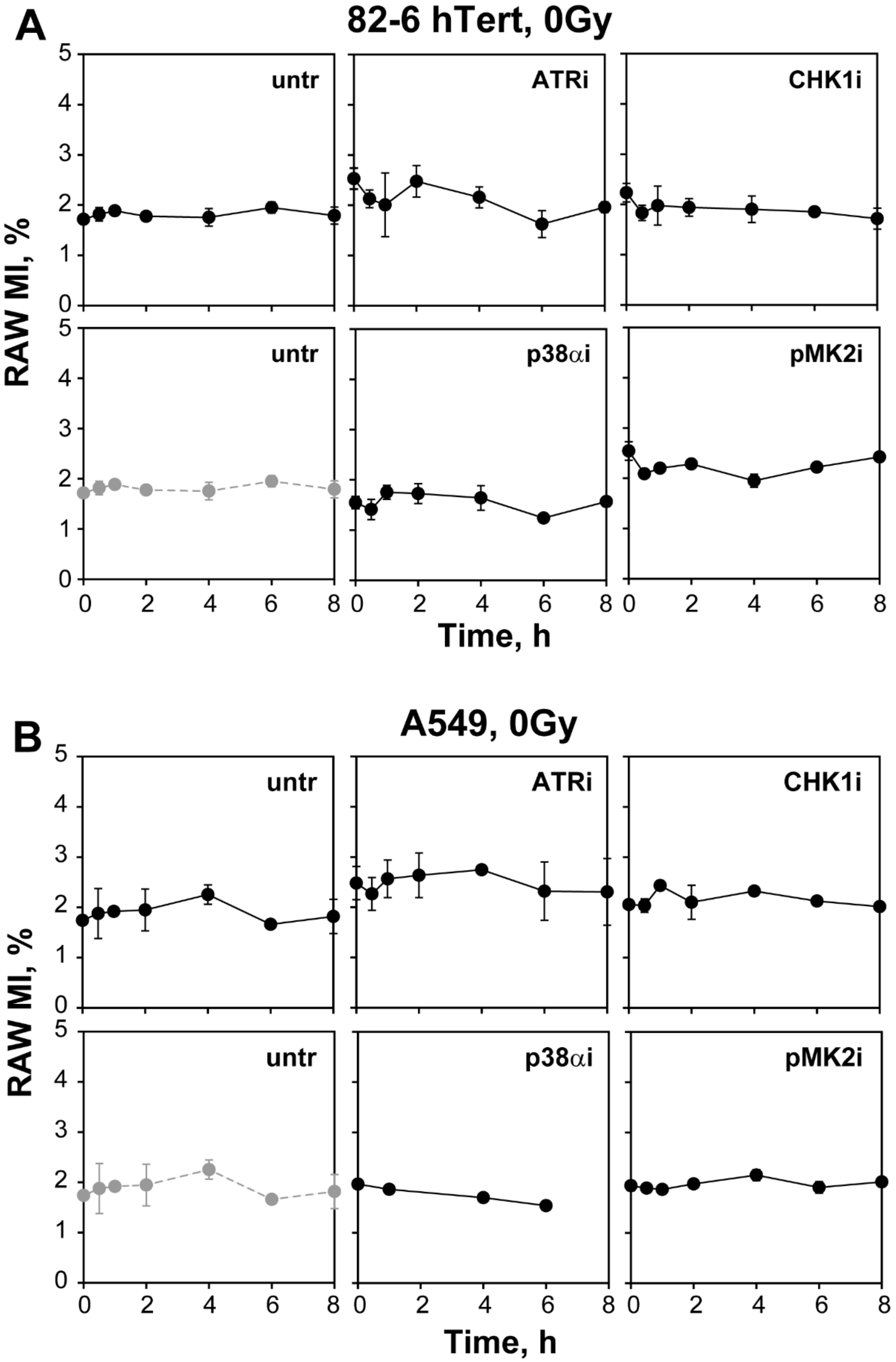
A
82-6 hTert, 0Gy
untr
ATRi
CHK1i
untr
p38αi
pMK2i
RAW MI, %
Time, h
B
A549, 0Gy
untr
ATRi
CHK1i
untr
p38αi
pMK2i
RAW MI, %
Time, h

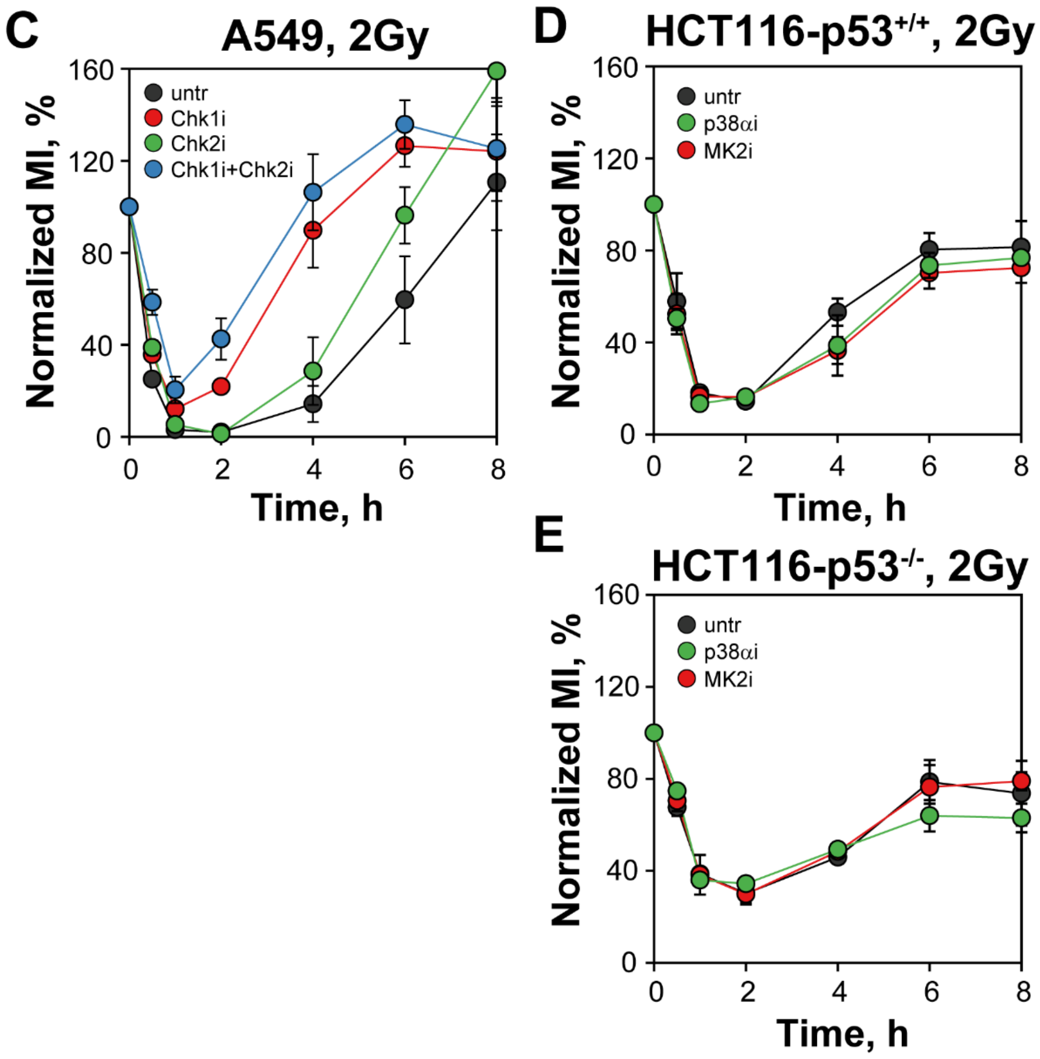

Figure S2. (A and B) RAW mitotic index of non-irradiated 82-6 hTert and A549 cells, treated with the indicated inhibitors. **(C)** As in Figure 1B for A549 cells exposed to 2 Gy in the presence of the indicated Chk1 and Chk2 inhibitors alone or combined. **(D)** Same as in panel C for HCT116-p53$^{+/+}$ cells. **(E)** As in panel D for HCT116-p53$^{-/-}$ cells. Data represent the mean and standard deviation (± SD) from three independent experiments.

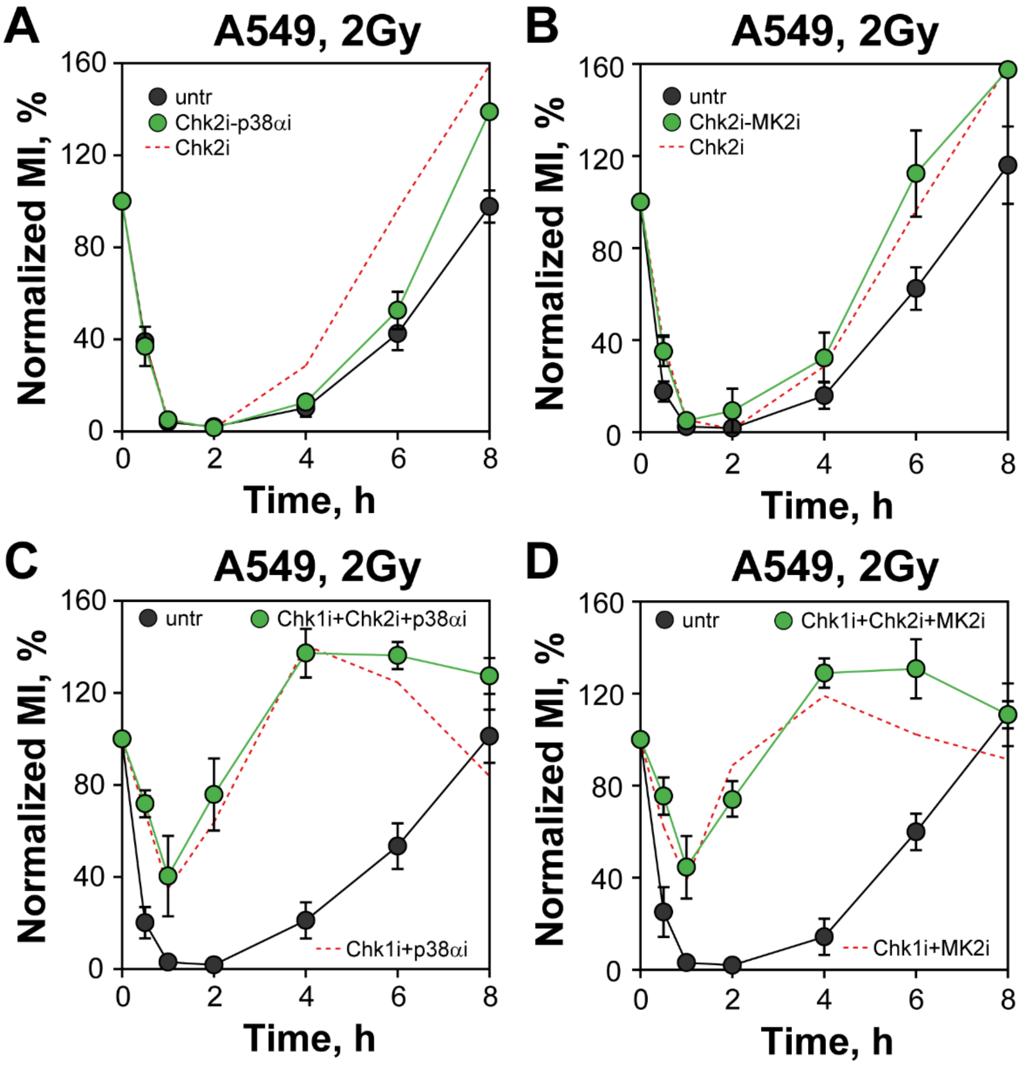

Figure S3. (A and B) A549 cells exposed to 2 Gy were treated or not with the indicated combinations of Chk2, p38αi and MK2i inhibitors and the MI was measured. Other details as in Figure 1A. The red dashed line depicts data of Chk2i treated cells from Figure S2A**. (C and D)** As in panels A and B but including Chk1i. The red dashed line depicts results shown in Figure 3B and 3D, respectively. All other details as in Figure 4. Data represent the mean and standard deviation (± SD) from three independent experiments.

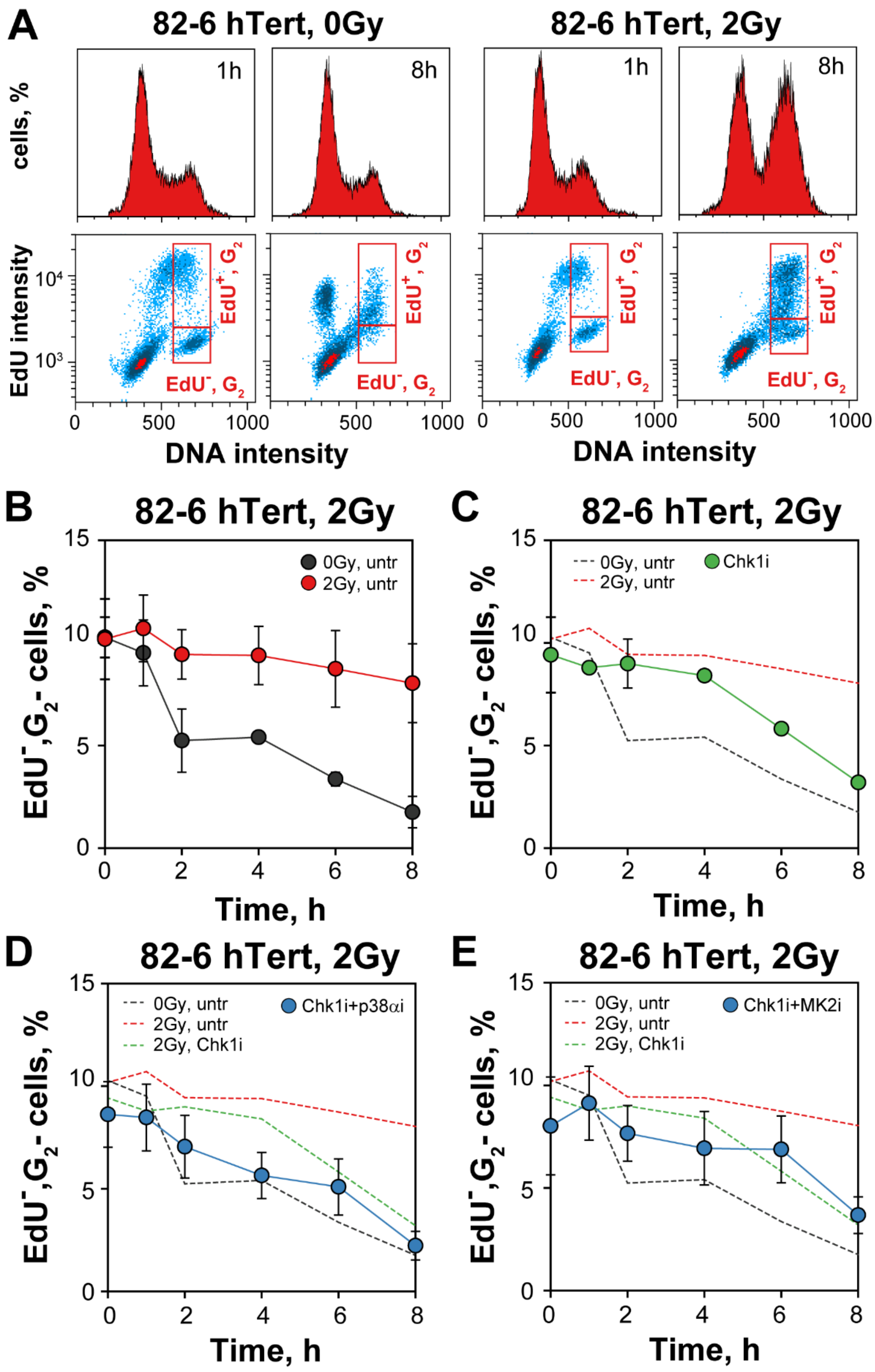
A
82-6 hTert, 0Gy
82-6 hTert, 2Gy
1h
8h
cells, %
EdU intensity
EdU⁺, G2
EdU⁻, G2
DNA intensity
B
C
D
E
82-6 hTert, 2Gy
0Gy, untr
2Gy, untr
2Gy, Chk1i
Chk1i
Chk1i+p38αi
Chk1i+MK2i
EdU⁻,G2- cells, %
Time, h

Figure S4. (A) Three-parameter flow cytometry analysis for combined detection of cells irradiated in S-phase for analysis after they progress into G_2-phase (EdU^+, G_2-cells), as well as of cells irradiated in G_2-phase and analyzed exclusively in G_2-phase - by excluding cells entering from S-phase during analysis (EdU^-, G_2-cells). PI-histograms (upper panels), illustrate the distribution of 82-6 hTert cells throughout the cell cycle in non-irradiated cultures or cultures exposed to 2 Gy. The examples of cell populations shown are collected 1 or 8 h after irradiation. A 30 min EdU pulse was utilized to label cells in S-phase and was applied just before radiation exposure. The gates used to select the desired cell populations are shown by red rectangles (lower panels). **(B)** Percentage of EdU^-, G_2-cells, in non-irradiated cells and cells exposed to 2 Gy, as a function of time after IR. **(C)** As in panel B in the presence of Chk1i. The dashed lines represent results shown in panel B and are included for comparison. **(D)** As in panel C for cells treated with both Chk1i and p38αi. The dashed lines, depict results from panels B and C and are shown for comparison. **(E)** As in panel D for cells treated with both Chk1i and MK2i. Other details as shown in panel D. Data represent the mean and standard deviation (± SD) from three independent experiments.

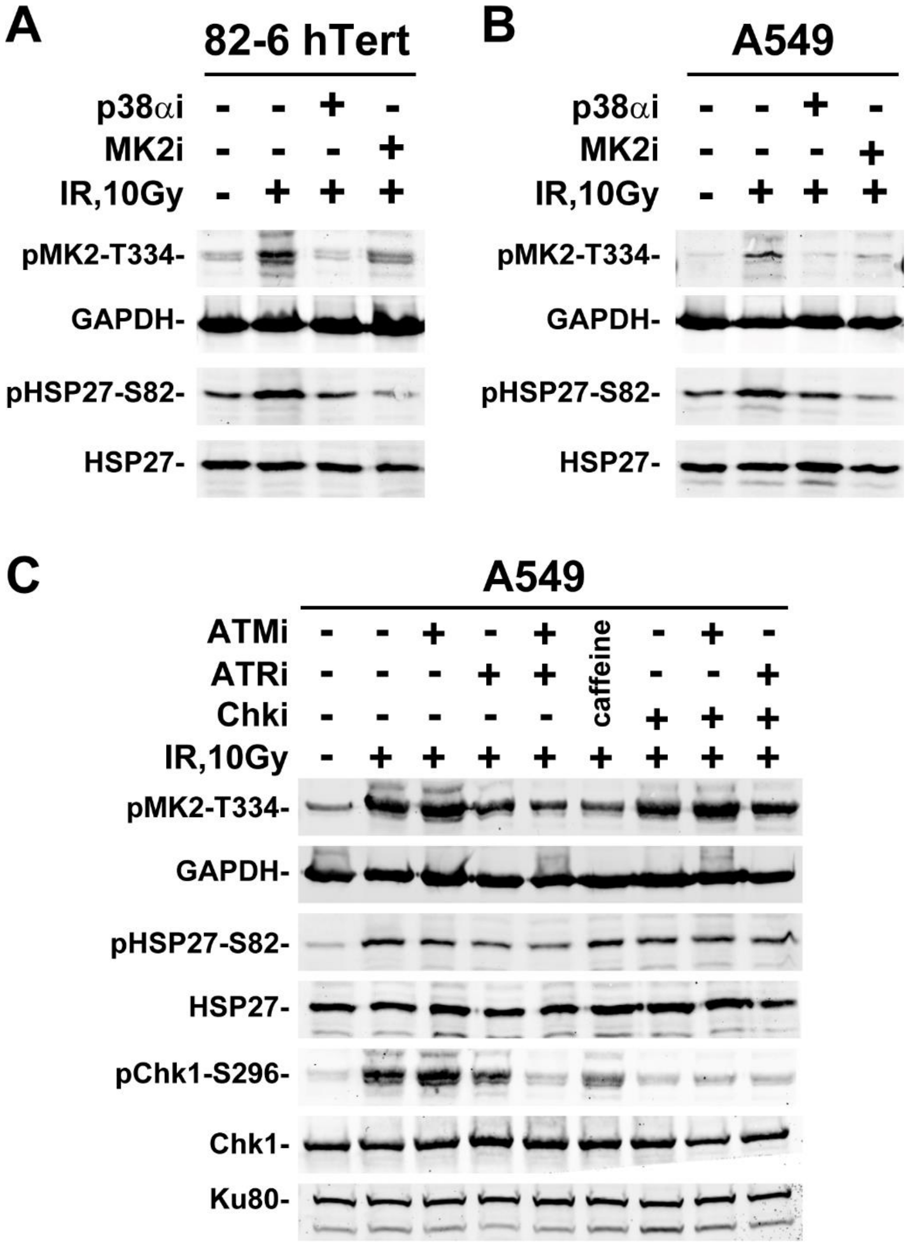

Figure S5. (A) Western blot analysis showing the activity of p38α after treatment with p38αi and MK2i of 82-6 hTert cells. The phosphorylation of MK2 at T334 was used as a proxy for the estimation of p38α activity, while the phosphorylation of HSP27 at S82 for the evaluation of MK2 activation. GAPDH and HSP27 served as loading controls. **(B)** Same as panel A for A549 cells **(C)** Western blot analysis of pMK2-T334 and pHSP27-S82 after treatment of A549 cells with the indicated PIKK inhibitors. GAPDH, HSP27, Ku80 and CHK1 served as loading controls.

Table S1

Compilation of utilized antibodies and dilutions employed.

Antibody name	Company	Clonality	Dilution
anti-Chk1 (G-4)	Santa Cruz Biotechnology	Mouse monoclonal	1:500
anti-pChk1-S345	Cell Signaling Technology	Rabbit polyclonal	1:500
anti-pChk1-S296	Cell Signaling Technology	Rabbit polyclonal	1:500
anti-HSP27	Cell Signaling Technology	Mouse monoclonal	1:500
anti-pHSP27-S82	Cell Signaling Technology	Rabbit polyclonal	1:500
anti-MK2	Cell Signaling Technology	Rabbit polyclonal	1:250
anti-pMK2-T334	Cell Signaling Technology	Rabbit polyclonal	1:250
anti-GAPDH	(MERCK, MAB374)	Mouse monoclonal	1:5000 to 1:10000
anti-XLF (D-1)	Santa Cruz Biotechnology	Mouse monoclonal	1:1000
anti-Ku80	GeneTex	Rabbit polyclonal	1:2000
anti-Ku70 (N3H10)	GeneTex	Mouse monoclonal	1:2000
anti-H3-pS10	Abcam PLC.	Rabbit polyclonal	1:1000 to 1:5000

RAW Western blot analysis data

The p38/MK2 pathway functions as Chk1-backup downstream of ATM/ATR in G_2-checkpoint activation in cells exposed to ionizing radiation

Luo et al., Cells

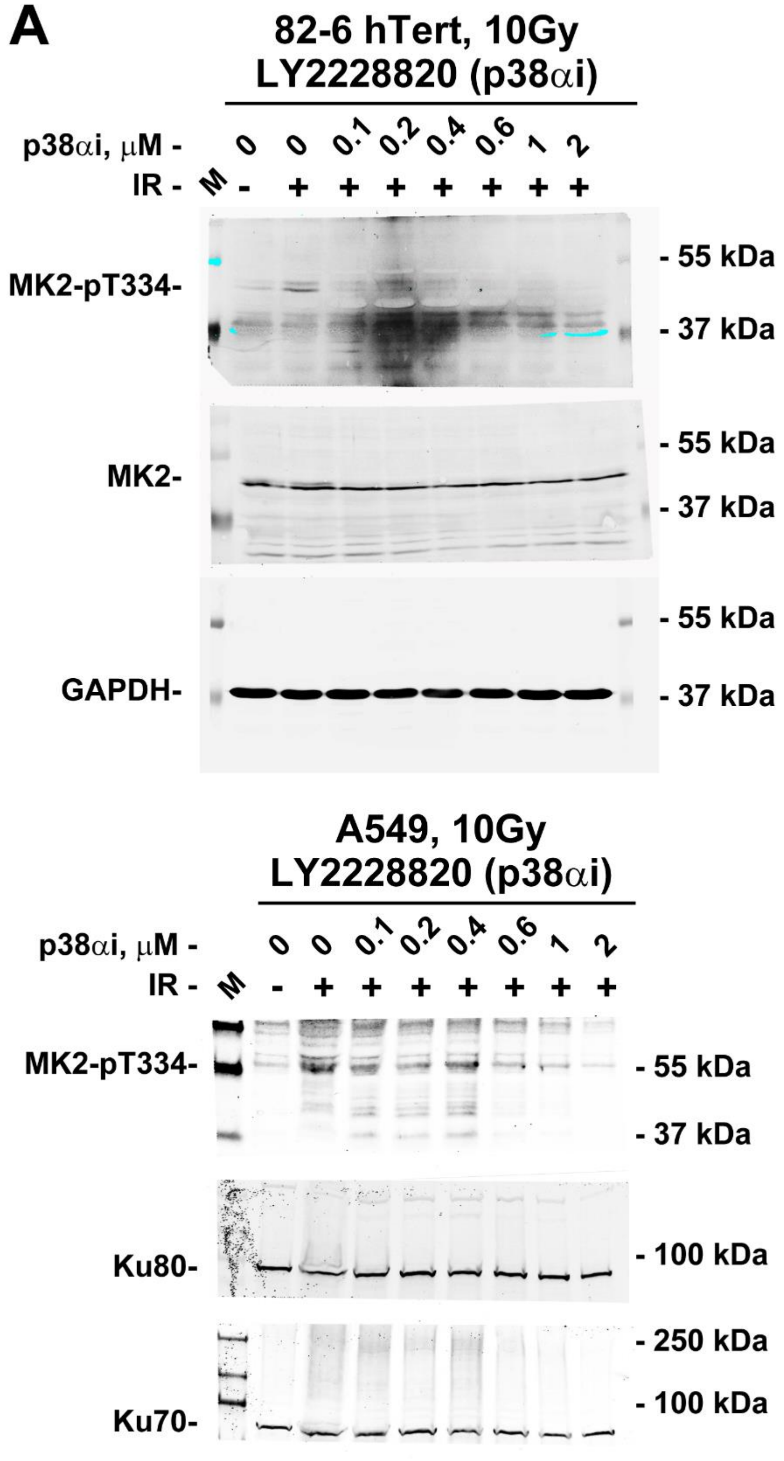

Luo et al. Raw WB data of experiments presented in Figure 2A. (M), indicates protein marker.

Luo et al. Figure S6

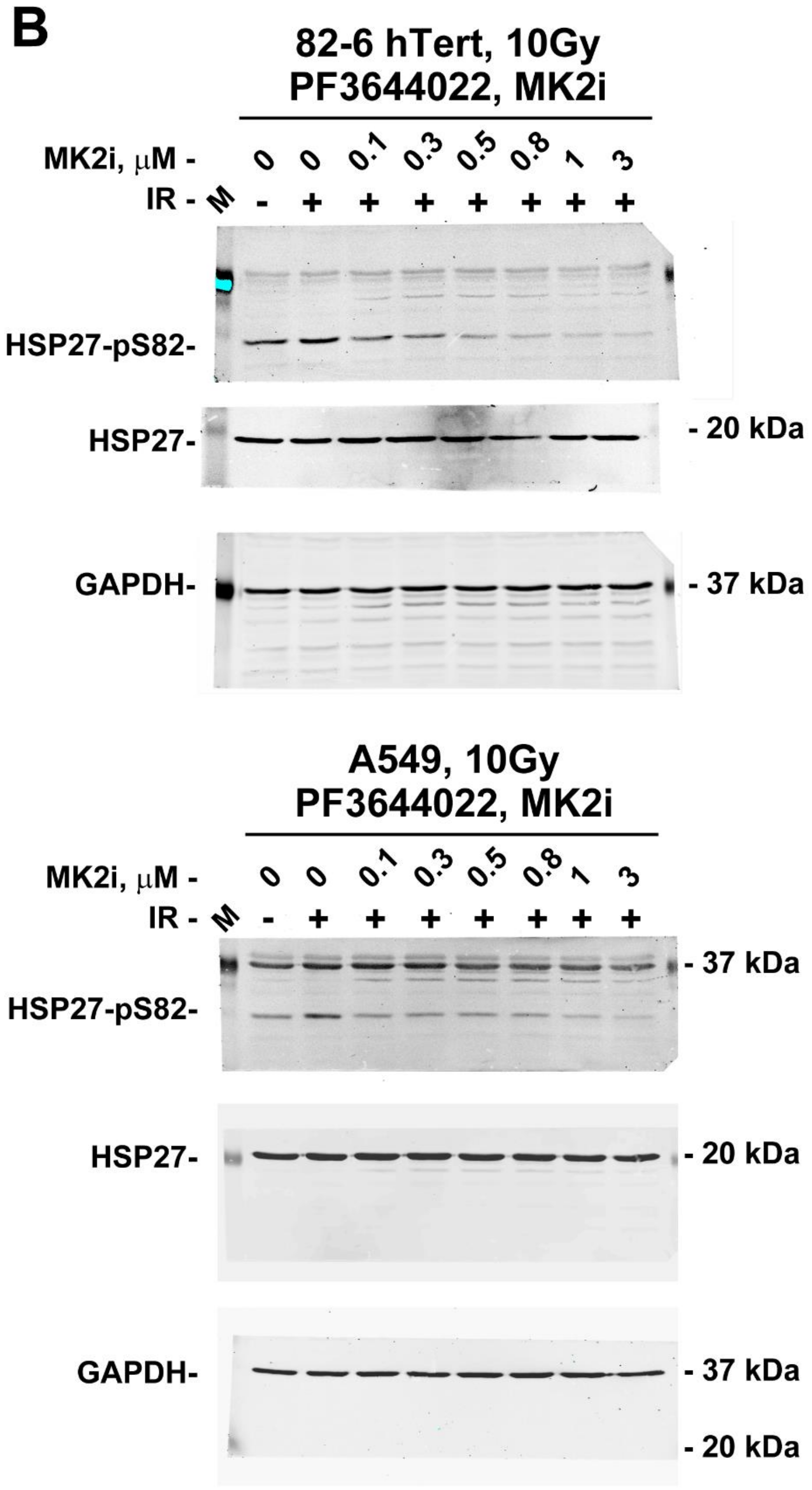

Luo et al. Raw WB data of experiments presented in Figure 2B. (M), indicates protein marker.

Luo et al. Figure S6

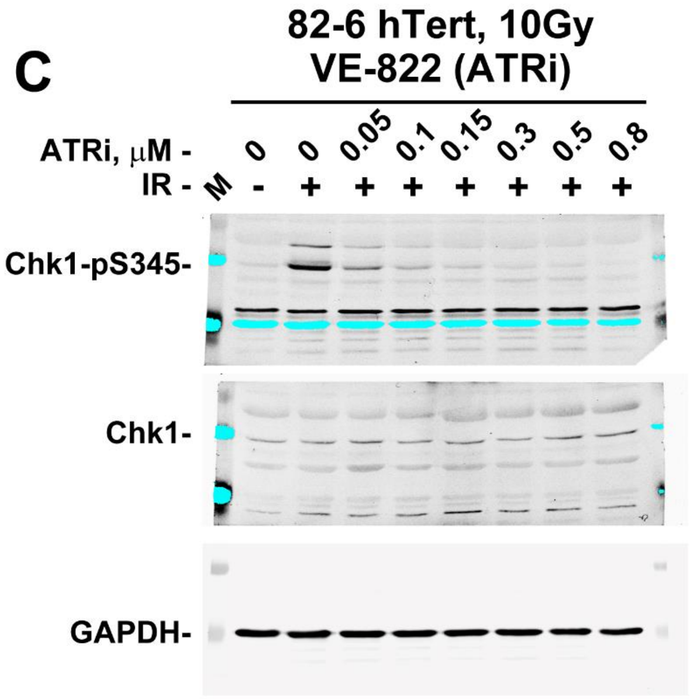

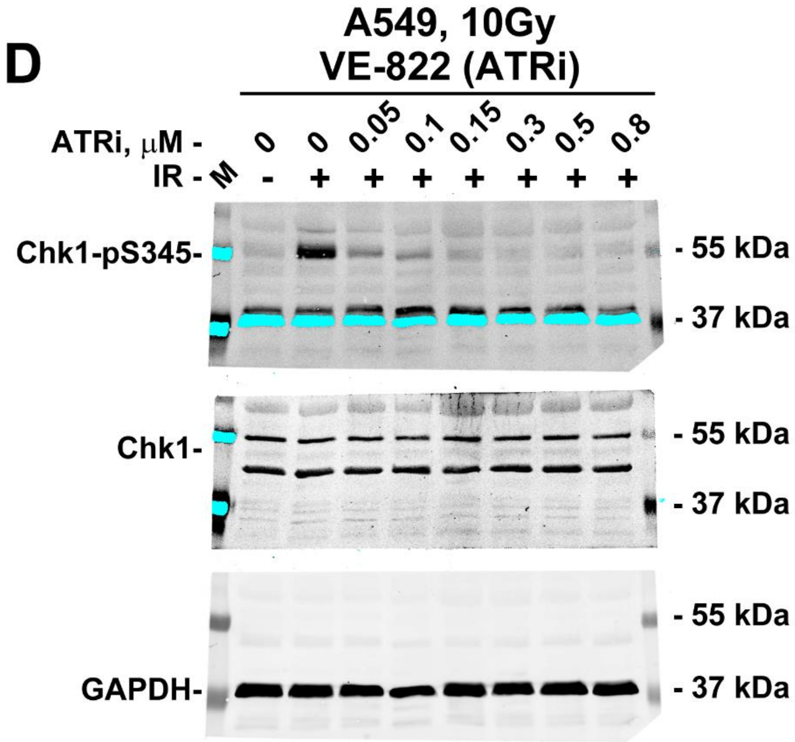

Luo et al. Raw WB data of the results presented in Figures S1C and S1D, (M), indicates protein marker.

Luo et al. Figure S7

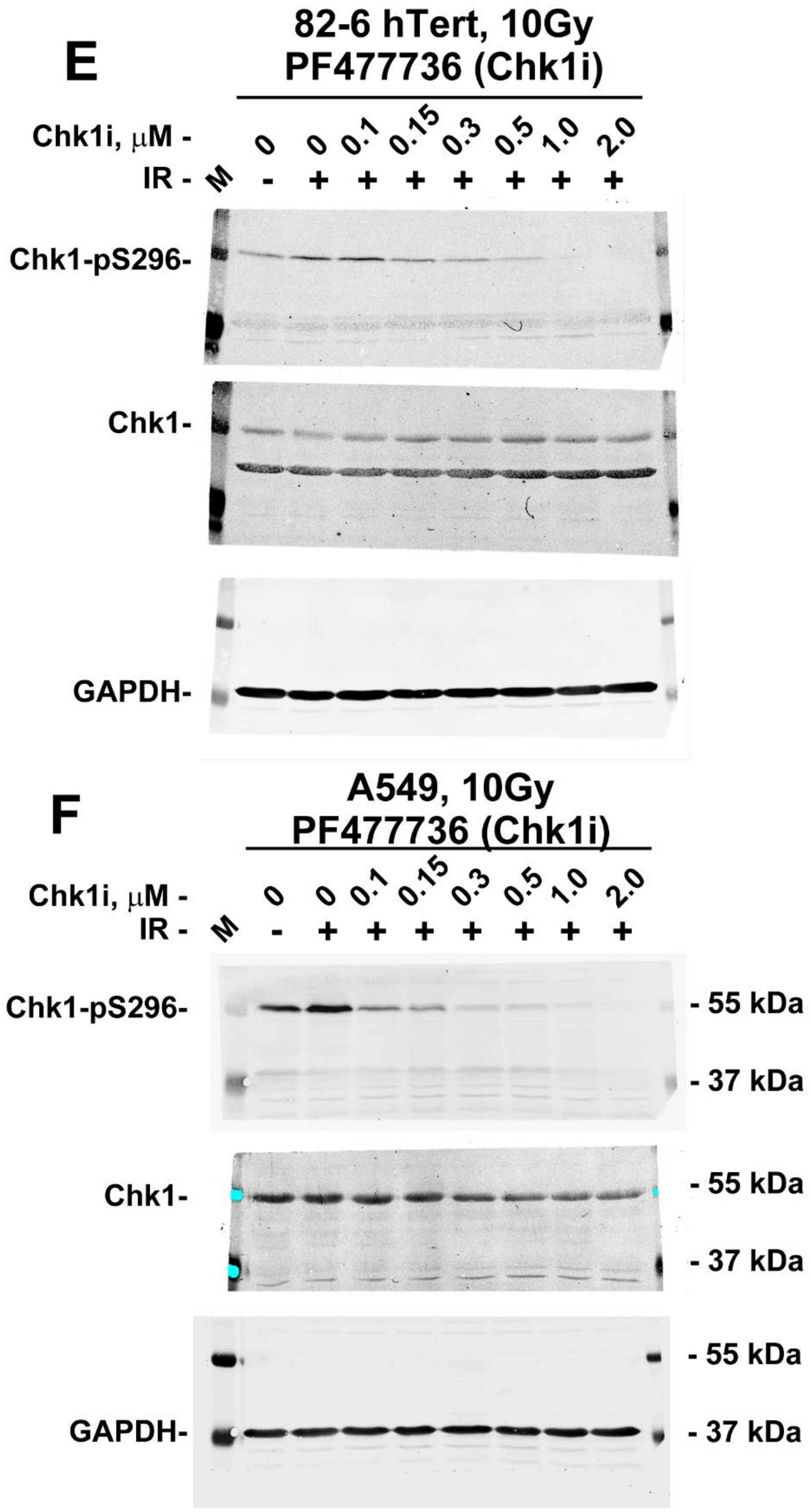

Luo et al. Raw WB data of the results presented in Figures S1E and S1F.
(M), indicates protein marker.

Luo et al. Figure S7

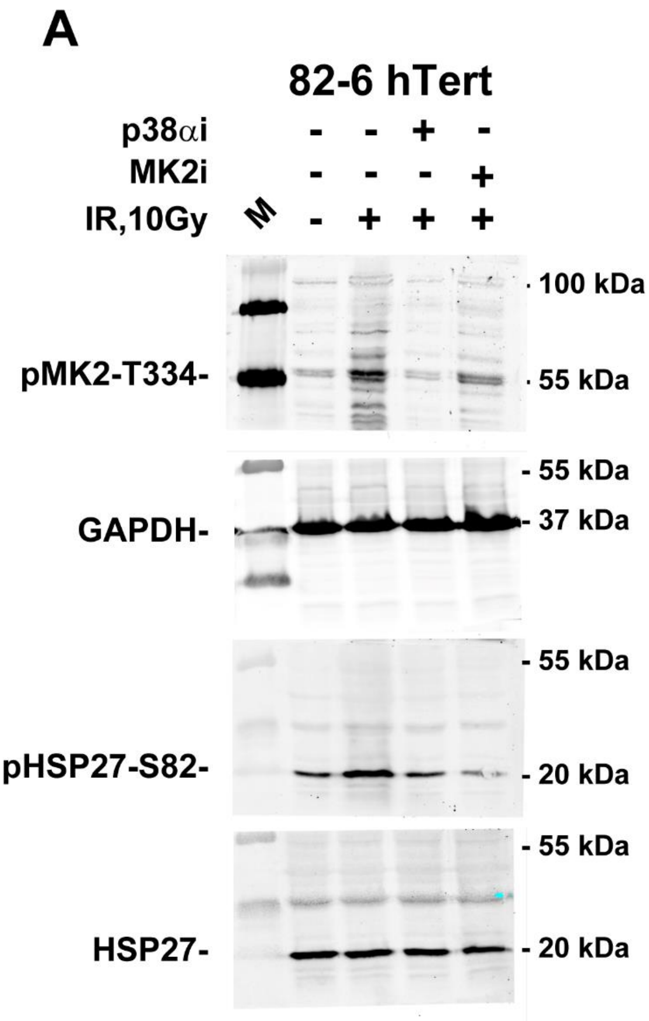

Luo et al. Raw WB data of the results presented in Figure S5A. (M), indicates protein marker.

Luo et al. Figure S8

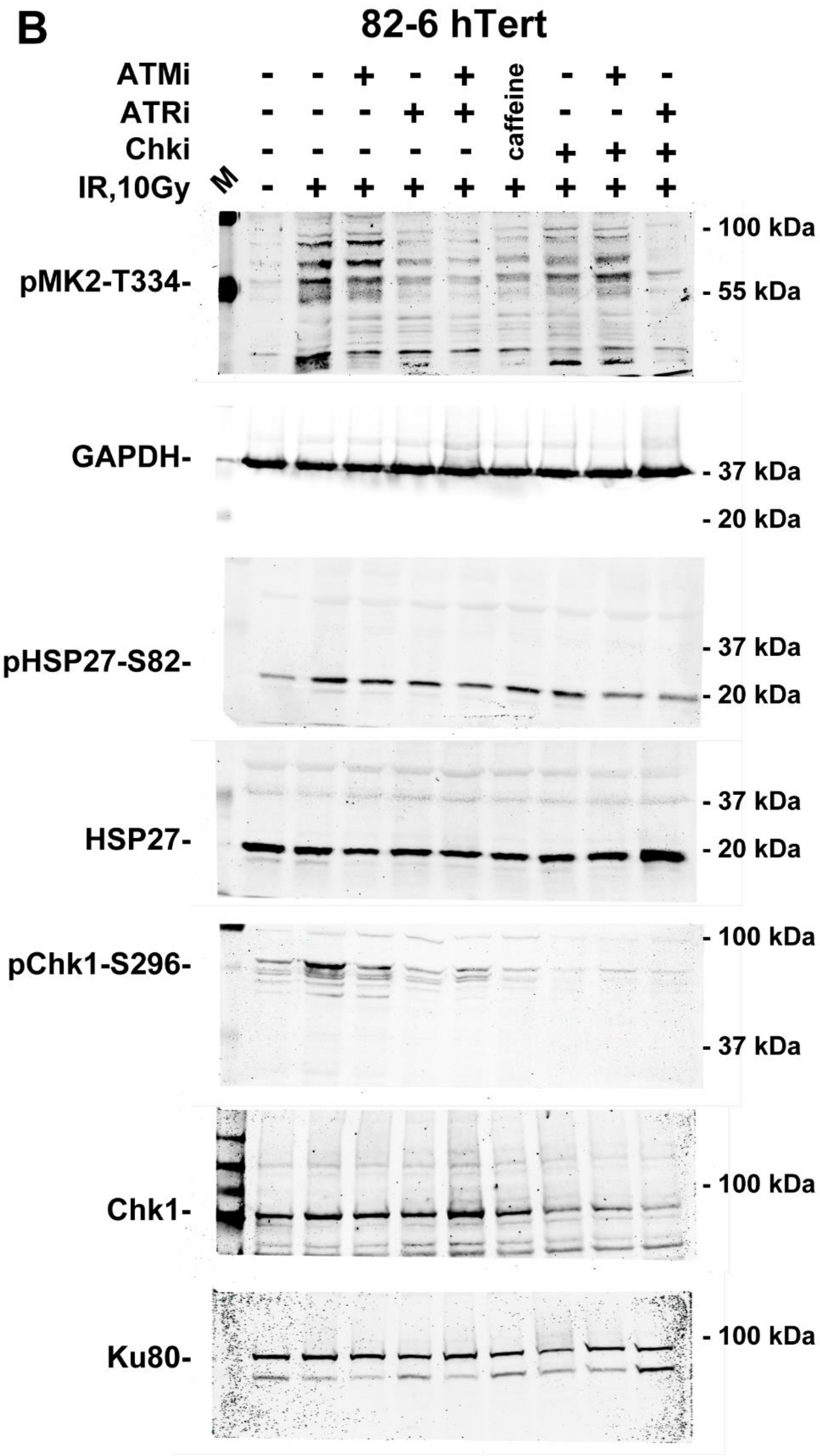

Luo et al. Raw WB data of the results presented in Figure 7A. (M), indicates protein marker.

Luo et al. Figure S8

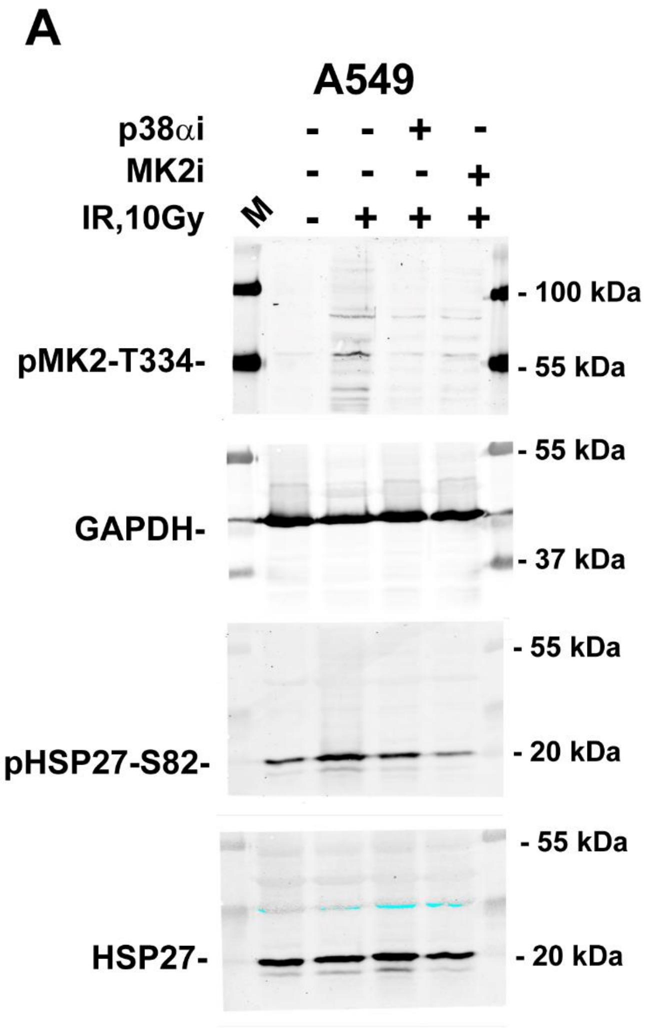

Luo et al. Raw WB data of the results presented in Figure S5B. (M), indicates protein marker.

Luo et al. Figure S9

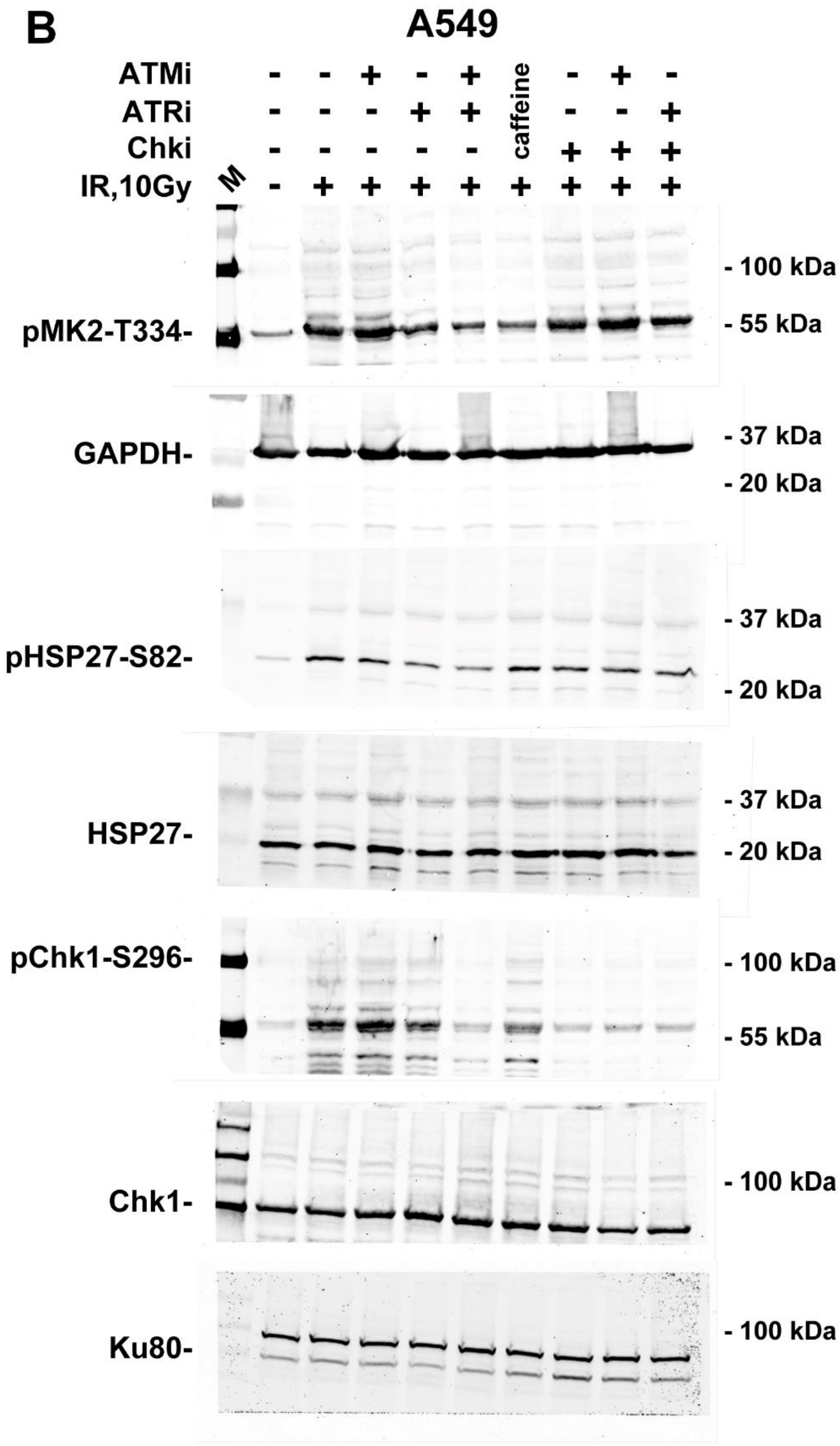

Luo et al. Raw WB data of the results presented in Figure S5C. (M), indicates protein marker.

Luo et al. Figure S9
